# A novel mechanism to generate metallic single crystals

**DOI:** 10.1038/s41598-021-04235-2

**Published:** 2021-12-29

**Authors:** J. Pistor, C. Körner

**Affiliations:** 1 Department of Materials Science and Engineering, Chair of Materials Science and Engineering for Metals, Friedrich-Alexander-Universität Erlangen-Nürnberg, Martensstr. 5, 91058 Erlangen, Germany; 2 Joint Institute of Advanced Materials and Processes, Friedrich-Alexander-Universität Erlangen-Nürnberg, Dr.-Mack Str. 81, 90762 Fürth, Germany

**Keywords:** Engineering, Materials science

## Abstract

Generally, the evolution of metallic single crystals is based on crystal growth. The single crystal is either produced by growing a seed single crystal or by sophisticated grain selection processes followed by crystal growth. Here, we describe for the first time a fully new mechanism to generate single crystals based on thermo-mechanically induced texture formation during additive manufacturing. The single crystal develops due to two different mechanisms. The first step is a standard grain selection process due to directional solidification, leading to a pronounced fiber texture. The second and new mechanism bases on successive thermo-mechanically induced plastic deformations and texture formation in FCC crystals under compression. During this second step, the columnar grain structure transforms into a single crystal by rotation of individual grains. Thus, the single crystal forms step by step by merging the originally columnar grain structure. This novel, stress induced mechanism opens up completely new perspectives to fabricate single crystalline components and to accurately adjust the orientation according to the load.

## Introduction

Single crystals from alloys or semiconductors are normally produced by crystal growth, e.g. the Czochralski method^1^, the Bridgman method^2,3^ or zone melting^4^ or by means of recrystallization as a consequence of abnormal grain growth^5^. Single crystalline components made from nickelbase superalloys are used in the hottest sections of stationary gas turbines or aircraft turbines^6^. Typically, these components develop in a Bridgman process where competitive grain selection during directional solidification and a geometric selection process (spiral selector) are combined to select one grain which eventually forms the component^7^. The primary orientation along casting direction of this selected grain is close to 〈001〉 direction whereas the secondary orientation is undefined. Since an exact orientation in 〈001〉 direction is impossible, deviations of about 15° are typically accepted by industry^8,9^. In addition, the dendritic solidification microstructure of these single crystals leads to inhomogeneities of the element distribution and solidification porosity on the scale of the primary dendrite spacing which is about several hundred microns^10–12^. For some alloys, even extremely long annealing times do not lead to homogeneous element distributions^11^.

In 2016 we were the first to demonstrate the single crystal formation by additive manufacturing (AM), using a conventional nickelbase superalloy^13^. Due to the inherent high solidification velocities, combined with steep thermal gradients, the dendrite arm spacing is about two orders of magnitude smaller compared to the classical Bridgman process^14,15^. As a result, these single crystals are homogenized within minutes^14^ and also the solidification porosity scales with the dendrite arm spacing^16^.

These additively manufactured single crystals show an unprecedented level of homogeneity which cannot be reached with conventional methods and which leads to superior properties such as the high temperature strength^17^, oxidation resistance^18^, creep strength^17^ or fatigue life^16^.

Until now, the basic mechanism leading to the formation of a single crystal during additive manufacturing was not clear. The goal of this paper is to elucidate the relevant mechanisms for single crystal formation in additive manufacturing. Based on this new insight, the potential of additive manufacturing to fabricate single crystalline components manifests.

## The texture of additively manufactured single crystals

Normally, texture formation, as a consequence of a solidification process, is strongly correlated with the direction of the thermal gradient. The mechanism of texture formation is based on grain selection by overgrowing during non-equilibrium solidification. In cubic crystal structures, grains with the smallest deviation between their 〈001〉 orientations and the thermal gradient will overgrow less well oriented ones by forming secondary dendrite trunks perpendicular to the solidification direction^19–21^. This strong [001] texture formation along the solidification direction is exploited during directional solidification according to the Bridgman process^6^. By applying an additional spiral grain selector, less well oriented grains will be blocked and ideally only one grain survives and forms a single crystal^7^. Nevertheless, the primary orientation of these single crystals still shows some deviations from the [001] direction and the secondary orientation cannot be controlled at all^7^.

**Figure 1:**
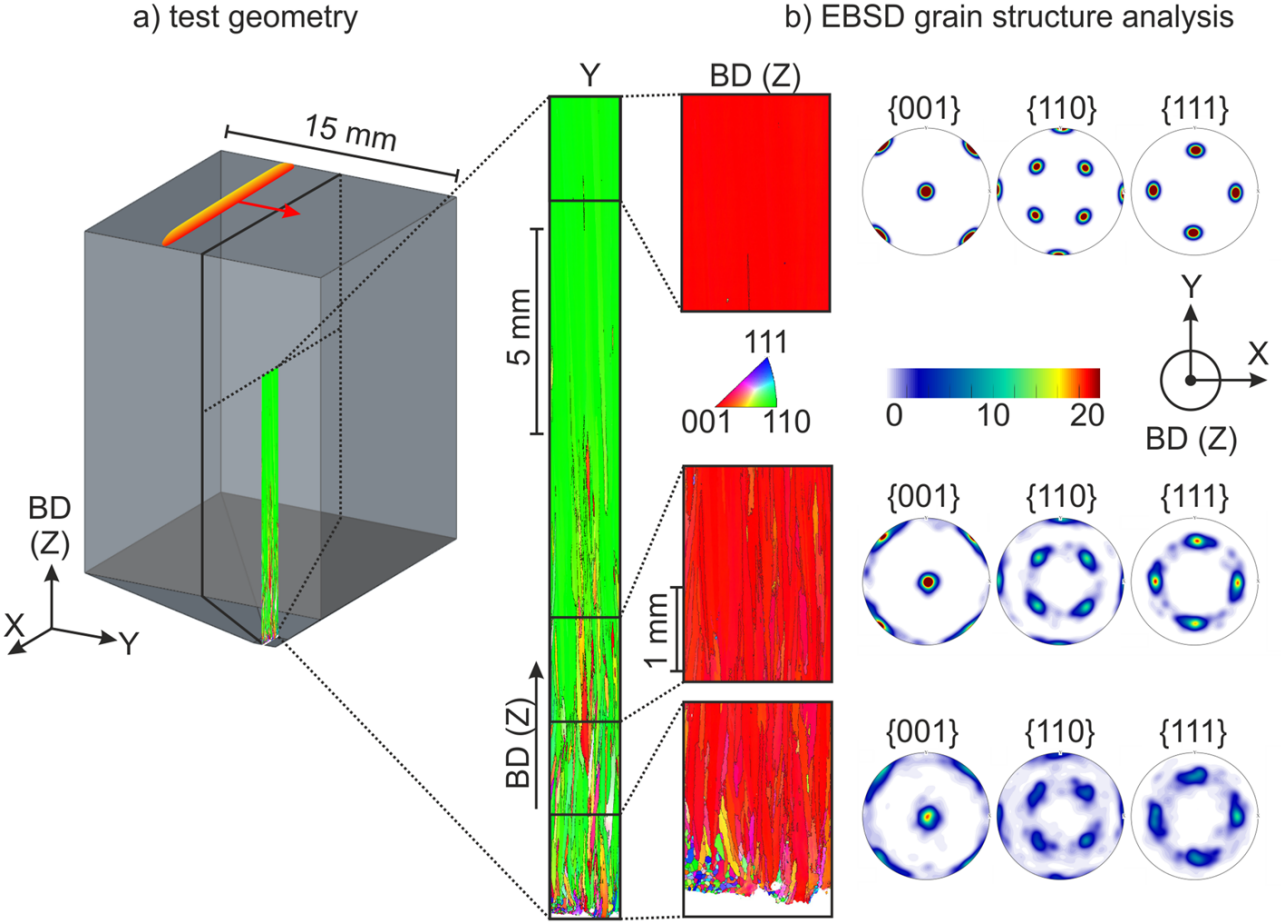
Texture evolution of AM single crystals. (**a**) Schematic of a test specimen. (**b**) EBSD grain structure analysis. The AM single crystals emerge from an initial columnar grain structure with distinct [001] texture along build direction Z (red) and [110] texture along melt direction Y (green).

The single crystals considered in this study emerge in a powder bed and electron beam based additive manufacturing process without using a single crystalline substrate material as seed. The additive build process starts on a poly crystalline substrate material. Directional solidification and grain selection processes lead very quickly to an initial columnar microstructure with pronounced [001] fiber texture along build direction BD (Z-direction). With proceeding build height, a single crystal emerges with defined primary (along build direction) as well as secondary dendritic orientation (in build plane, Y-direction), see Fig. 1.

A typical cuboid test specimen is displayed in Fig. 1a). Under standard building conditions with a trailing melt pool (i.e. the movement of the melt pool follows the movement of the beam) single crystals do not evolve. In a former study we have experimentally shown that one prerequisite for single crystal evolution is the presence of a persistent line shaped melt pool^22^. The melt pool shape induced by the beam and its lateral velocity is indicated on the surface of the sample. In this case, the movement of the beam in X-direction is perpendicular to the movement of the melt pool in Y-direction.

To investigate the microstructure evolution, cross sections of the sample are prepared along the build direction according to the cutting plane in Fig. 1a) and EBSD measurements are performed in the center (Fig. 1b). A strong primary [001] orientation is observed, making the inverse pole figure (IPF) coloring along build direction (BD) appear red. At a building height of 15 mm, the deviation between the [001] direction and BD is about 1° or 2°, much less compared to cast single crystals^23^. In addition, the secondary orientation shows a pronounced [110] texture along the melt direction, which makes the IPF Y maps appear green. It is important to notice that the [010] orientation of the dendrites is twisted by 45° with respect to the melt pool direction along Y-direction. This observation is fundamental and was first described by Ramsperger et al. in 2016, but could not be explained at that time^13^. The already published approaches to explain single crystal development by a µ-helix selection process induced by the gradient of the temperature field^24^ or by taking into account the shape of the melt pool^25^ are also not able to explain the experimental findings. The harsh solidification conditions lead to nearly cellular dendritic growth. Thus, excessive side branching during solidification cannot be observed in a sufficient amount for a grain selection mechanism based on overgrowing in additive manufacturing. Obviously, the underlying mechanism here is different from that during the Bridgman process.

The observation of the strong [110] texture along the melt direction is the key factor to reveal the working mechanism. This texture cannot be explained based on the classical grain selection process based on overgrowing under a directional thermal gradient. In contrast, from classical theory considering the thermal gradients, we would expect a clear [010] texture in the build plane as the gradient is aligned along the melt direction^26^. Thus, some effect is yet missing to explain our experimental findings.

Generally, texture not only develops by grain selection but also by plastic deformation e.g. in a rolling process^27,28^. During additive manufacturing extremely high stresses are induced by the local energy input, leading to plastic deformation. In the following, we will show that these stresses and the inherent strains are the origin of the development of AM single crystals with nearly perfect orientation.

**Figure 2:**
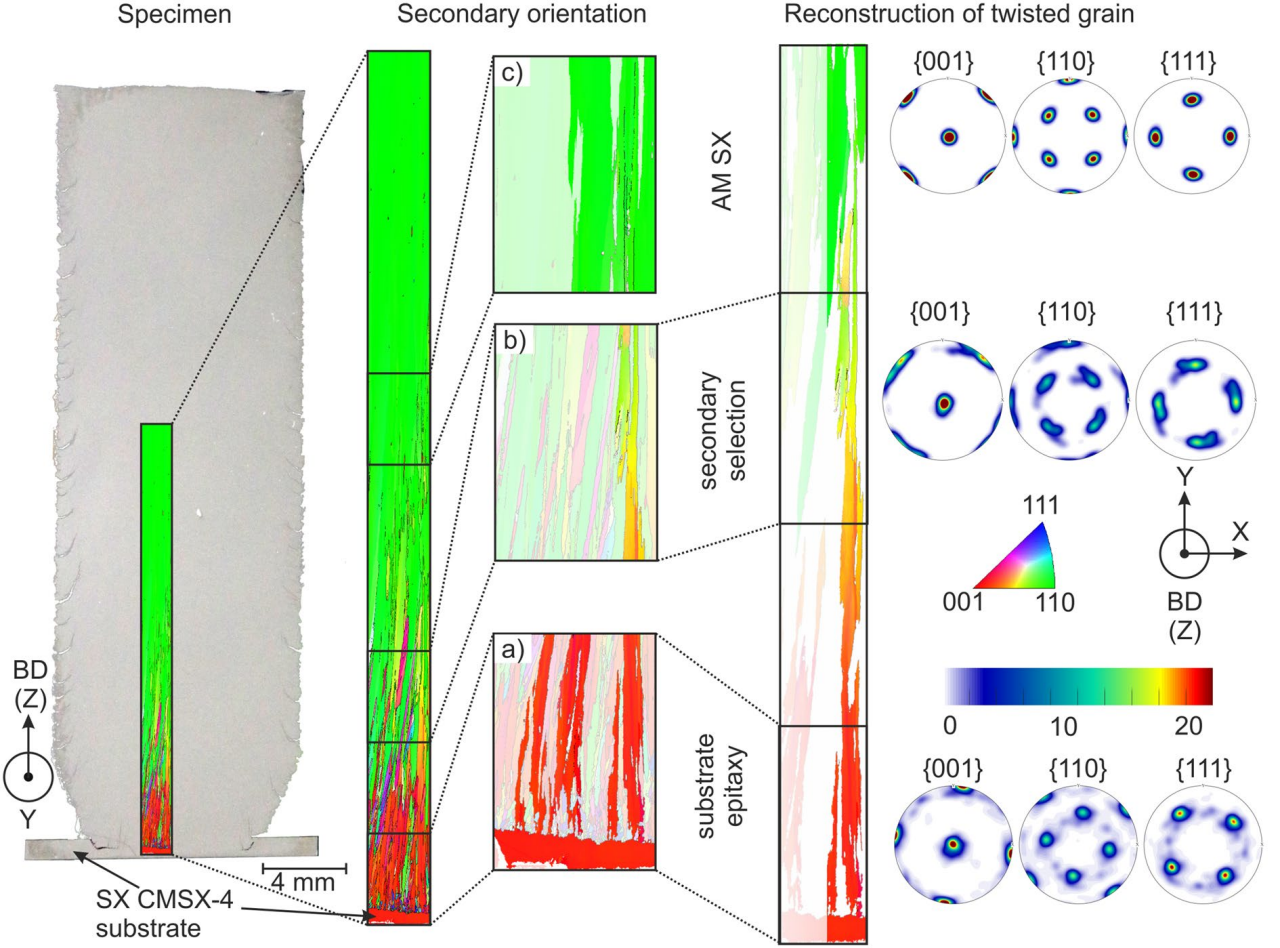
AM SX cylindrical build up on cast CMSX-4 SX substrate with different secondary orientation (left). Initial epitaxial columnar growth with the [100] substrate secondary orientation can be observed in the AM SX (middle). However, after a few millimeters the [110] orientation emerges more distinctly, until a newly [110] single crystal is formed after around 10–15 mm. All the grains show a gradual change of color in the IPF-Y maps towards [110]. One grain (right) was detected that is originated in the [100] substrate and ends in the [110] AM SX whereas it presents a continuous twist while being completely visible in the cutting plane. All columnar grains will homogeneously deform towards [110] and merge into a single crystal. The pole figures represent the texture in the three positions: Substrate epitaxy (**a**)), secondary selection (**b**)) and AM SX (**c**)).

In order to elucidate the mechanism for the formation of the [110] texture we performed a fundamental yet simple experiment where we used a single crystalline substrate material (CMSX-4) closely oriented with [010] along melt direction, see Fig. 2. Starting with a single crystal, epitaxial growth with [010] secondary orientation was expected. However, with increasing height the secondary orientation changes gradually from [010] to [110]. After 10–15 mm a newly selected additively manufactured single crystal with homogeneous [110] secondary orientation is formed. That is, even if a defined orientation is induced through the start plate material, the grain structure will eventually end up as [110] according to the applied scanning strategy. In order to reveal the reason for this behavior we investigated in more detail the grain structure and noticed that the orientation of individual grains is not constant but changes with building height. For the IPF-Y orientation, we observed a gradual color change within the columnar grains from red [010] towards green [110] whereas the IPF-BD orientation remains constant and close to [001]. That is, the grains are continuously twisted around the BD-axis until they reach the [110] direction after 10–15 mm. Figure 2 demonstrates this effect by considering a distinct grain, identified according to a 10° misorientation criterion, initiating in the close to [010] oriented SX substrate and ending in the [110] AM single crystal. This twisting effect is obvious in the IPF-Y maps as well as in the respective pole figures at positions (a)–(c) and ends when the [110] orientation is reached. During this process, the misorientation between the present columnar grains reduces continuously and the high angle grain boundaries transform into small angle grain boundaries (misorientation < 10°–13°^8,9^) and eventually vanish more and more with increasing build height. Nevertheless, the initial columnar grain structure is often still visible as sub grain structure in the AM SX material^23^. In contrast to casted single crystals that develop by the selection and growth of one grain, AM single crystals develop by a two-step process, namely through development of a fiber texture by grain selection coupled with some mechanism to merge these columnar grains into a single crystal.

## A thermo-mechanical approach to texture formation

**Figure 3:**
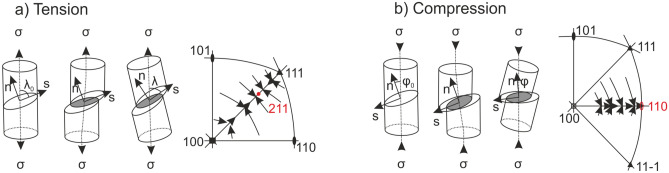
Schematic drawing of the texture formation in FCC crystals under tension (**a**) and compression (**b**) that leads to distinct rotation towards 〈211〉 under tension and to 〈110〉 under compression^27,28,30^.

In order to understand the origin of the observed grain twisting, resulting in single crystal formation, it is instructive to consider the well-known behavior of face centered cubic (FCC) crystals under compression or tension load, see Fig. 3. Plastic deformation is mainly accomplished by glide processes on the 12 glide systems 〈110〉 {111} according to Schmid’s law, once the resolved shear stress τ_C_ exceeds a specific critical value for the individual glide system^29^.

$${\tau }_{C}= \pm {\sigma }_{X}\,\mathrm{cos}\,\lambda\, \mathrm{cos}\,\phi $$

where σ_X_ is the normal stress, λ describes the angle between σ_X_ and the glide direction s and φ is the angle between σ_X_ and the glide plane normal n. During loading, the crystal rotates and aligns its 〈211〉 direction in tensile loads^27,28,30^ and the 〈110〉 direction under compressive loads^27,28^ along the load axis according to the schematics in Fig. 3a,b. That is, under compression deformation, FCC crystals will align their 〈110〉 direction in load direction. If this mechanism is responsible for the experimental observation, strong anisotropic compression stresses inducing plastic deformation have to be present during AM. In addition, these compressive stresses inducing plastic deformation have to be parallel or perpendicular to the beam velocity. That is, we have to analyze the stress and strain fields during the building process.

From experimental observation, we know that only specific process parameter combinations lead to single crystals^22^. In contrast to classical AM process parameters where the melt pool follows the beam (trailing melt pool), SX parameters are characterized by persistent, line-shaped melt pools^22^. The extension of these melt pools is parallel, the movement perpendicular (red arrow in Fig. 1a)) to the beam velocity, i.e. the scanning direction X. In addition, the longitudinal extension (X-direction) of the melt pool has to be much larger than the lateral one (Y-direction). This experimental observation is important since it already gives some hint that a symmetry breaking energy deposition is the key to fabricate single crystals. Anisotropic temperature fields will also lead to anisotropic stress states. Do these stress fields also generate the compressive stresses and plastic deformations necessary for the formation of the texture?

**Figure 4:**
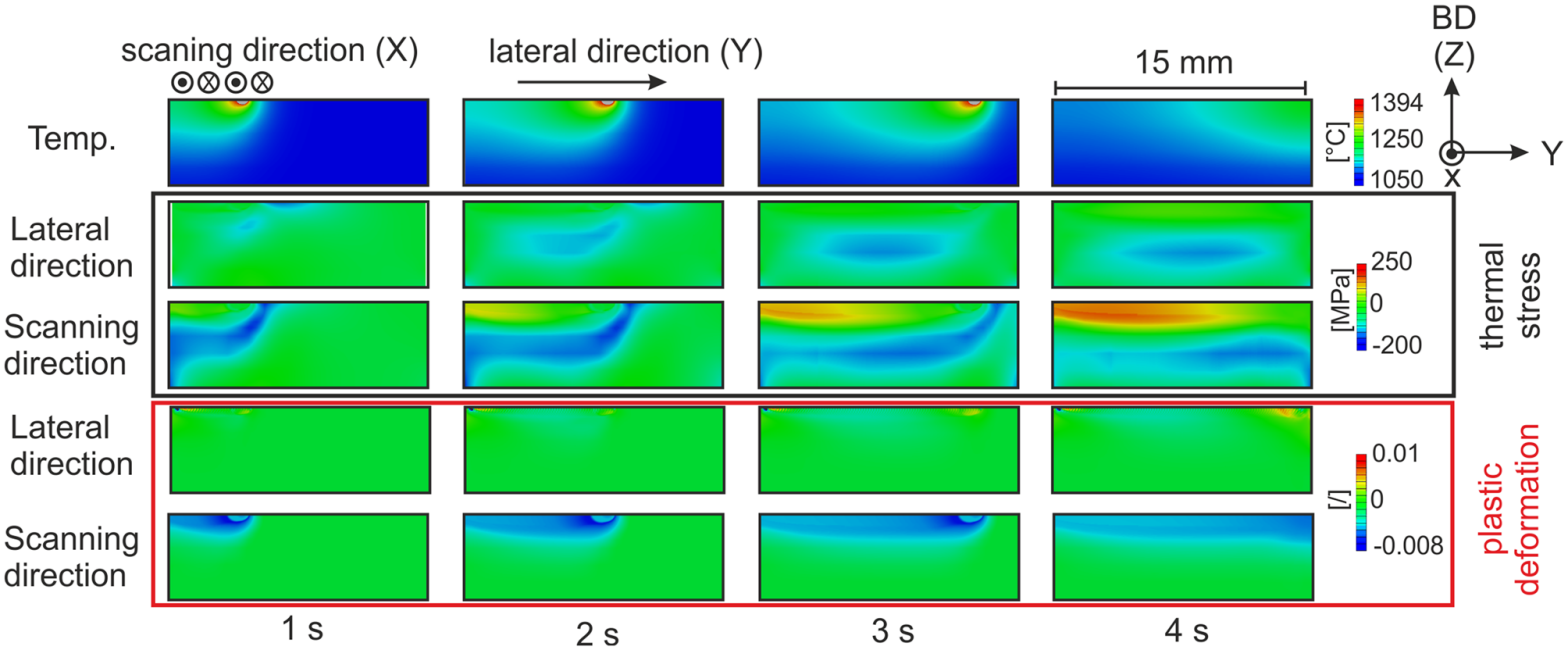
Finite element simulation of the temperature field and emerging thermal stresses by melting with a persistent melt pool leading to the SX structures in Fig. 1. A distinct and highly anisotropic compressive stress field is generated underneath the melt pool during melting. A spatial tensile stress field is generated near the surface with progressing melting. Anisotropic plastic deformation occurs mainly in scanning direction (X) according to the induced thermal compressive stresses.

In order to investigate the thermal induced stress and strain fields during melting and solidification with a persistent melt pool we performed a finite element simulation with the SX parameter set and sample geometry from Fig. 1. The temporal evolution of the temperature, stress and plastic strain fields in lateral direction and scanning direction are depicted in Fig. 4. Generally, the stress and strain fields are highly anisotropic. The absolute values are much higher in longitudinal X-direction compared to the lateral Y-direction, the stresses and strains along build direction (Z) are much lower and not depicted in Fig. 4. A compression stress field in the neighborhood of the melt pool is present as a consequence of the temperature field during the melting process. These stresses partly relieve by plastic deformation, resulting in negative and strongly anisotropic plastic strains near the surface, whereas tension stresses develop near the surface as result of this plastic deformation. Plastic deformation is mainly in the X-direction, i.e. the direction of the movement of the beam. In fact, the simulation predicts an anisotropic plastic deformation of the material under compression stresses as consequence of the induced thermal stresses. According to texture formation of FCC crystals under compressive loads, the FCC crystal structure is expected to develop a distinct [110] texture in the longitudinal X-direction. For grain rotation in the build plane (X–Y-plane), only four out of twelve glide systems of the FCC structure are considered relevant, which do not have a glide direction along [001]. When the secondary grain orientation is in [100] direction with respect to the scanning direction (i.e. the main direction of the compressive loads) all four glide systems are active as they present a Schmid factor of 0.408. The plastic deformations will lead to a rotation of the crystal lattice towards [110]. Once this orientation is reached all four glide systems are deactivated as their Schmid factor approaches zero and eventually a homogeneous [110] texture is formed. Hence, plastic deformation in the build plane (i.e. secondary orientation) can now only occur, if the stress field is oriented in a different way (i.e. by a change in scanning strategy). Due to symmetry considerations, the scanning direction is rotated by 90° in each layer, the simulation predicts exactly the texture evolution we have found during SX fabrication. The formation of the secondary orientation is based on a rotation around the primary [001] orientation (which is given by the thermal gradient) in the build layer and thus in accordance with the experimental observation of the grain twist in Fig. 2.

It is important to notice that the pronounced formation of the [110] texture along the scanning direction does not require a scan rotation at all. Experiments show that SXs appear at 180° change in each layer or 90° change in each layer. Nevertheless, the primary [001] texture in build direction depends on the scanning strategy. Only symmetric strategies result in a perfect [001] orientation in build direction, even sharper as expected solely due to effects of the thermal gradient and the correlated grain selection process. If the symmetry is broken, e.g. by only hatching in one direction, the related stresses lead to a deviation from this direction and the primary orientation [001] cannot be maintained. Furthermore, if the scan direction for subsequent layers changes by an angle between 0° and 90°, no SX develops as the secondary orientation [110] cannot be adjusted homogeneously throughout the build. Again, this demonstrates the role of the stress induced plastic deformation and the importance of symmetry to end up with perfectly oriented single crystals.

## The mechanisms of single crystal formation

**Figure 5:**
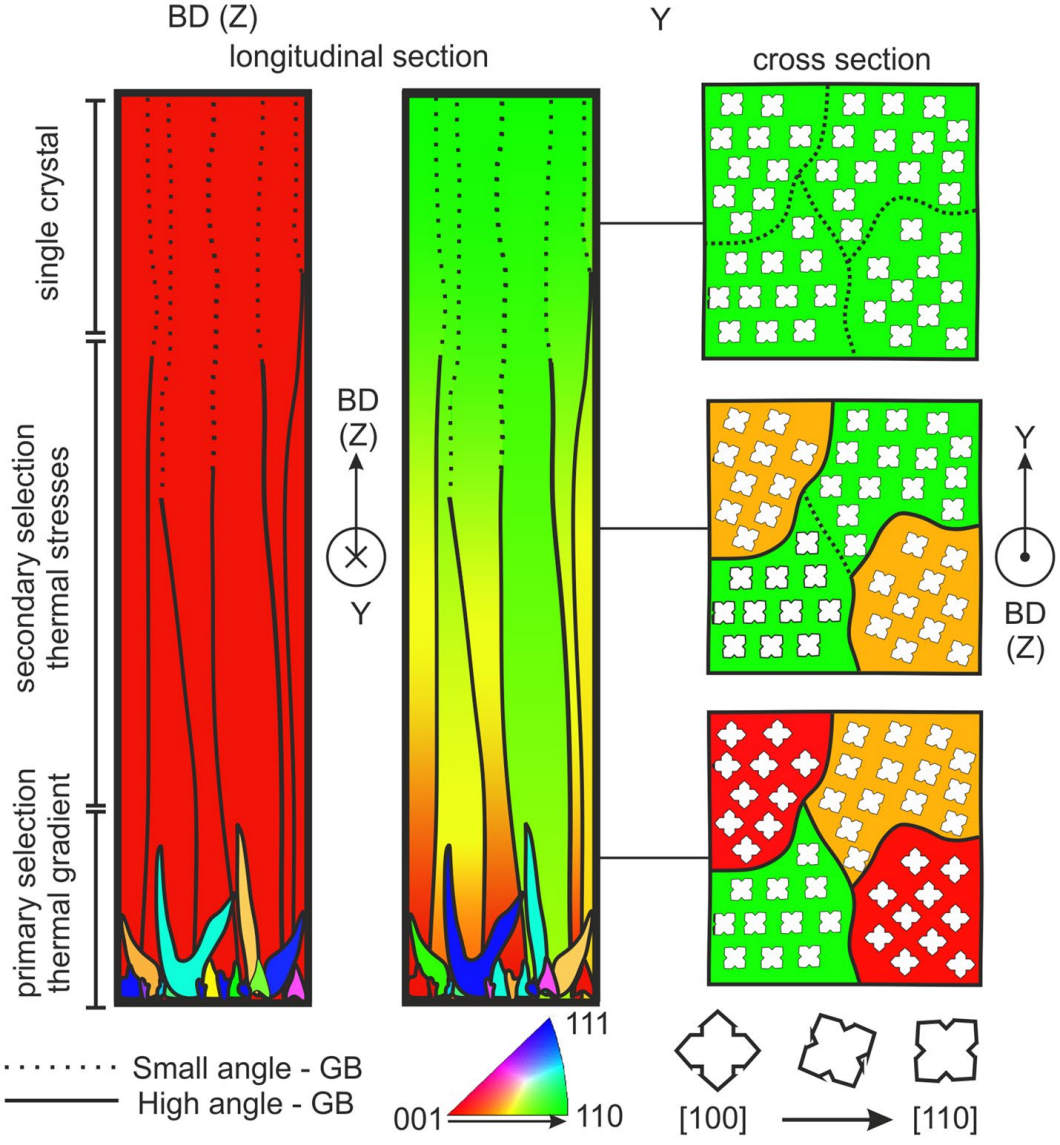
Schematic drawing of the two mechanisms leading to a single crystal in additive manufacturing. The thermal gradient based grain selection mechanism leads to competitive grain growth and a strong [001] texture along the build direction (BD). The thermal stress induced plastic deformation leads to the secondary orientation [110], the grains get twisted and high angle grain boundaries disappear as the grains merge together.

The presented results reveal a completely new mechanism for the fabrication of single crystals with unrivalled homogeneity and precision of orientation during additive manufacturing. The single crystal develops by a combination of grain selection and a thermo-mechanical induced merging of individual grains, see Fig. 5. The initial equiaxed and columnar grain structure undergoes a fast grain selection process based on competitive grain growth according to the steepest thermal gradient within the first layers, leading to a pronounced [001] fiber texture after about 500 µm along build direction. The resulting columnar grain structure presents a well-defined primary as well as an arbitrary secondary orientation with high angle grain boundaries (GB) between the individual columnar grains. Strong anisotropic stress fields induced by line shaped persistent melt pools progressively deform individual grains through twisting within the build plane towards the [110] secondary orientation along the Y direction. This progressive plastic deformation is visualized by the continuous orientation change of individual grains as a function of the build height.

Since the symmetry of the stress fields in single layers does not perfectly match the crystal symmetry, a scan rotation is necessary to achieve and maintain the single crystal. The layer wise scan rotation and the corresponding temperature and stress fields have to match the crystal symmetry (i.e. multiples of 90° for cubic). The formation of a single crystal is disrupted if the secondary selection is hindered by a scan rotation that is not compatible with the crystal symmetry (e.g. 67.5° for cubic) or once the primary selection is negatively influenced when the stress field is not compensated through a sufficient scan rotation (e.g. 0° rotation).

As the thermal stress field is active throughout the complete build in every single layer, the quality of AM single crystals with respect to misorientations between dendrites (mosaicity) is improving with build height. This is in contrast to the casting process where the grain selection is only done in the very beginning and the mosaicity is getting worse with progressing solidification^31^.

Moreover, with inducing a defined change in the thermal stress field after the successful formation of the single crystal, the single crystal can be deformed without forming high angle grain boundaries. The possibility to adjust both primary as well as secondary orientation according to the part geometry and also the controlled deformation of a single crystal may lead the way to a new generation of single crystal superalloys processed by additive manufacturing.

## Methods

### Additive manufacturing process

The spherical metal powder for the electron beam based powder bed fusion (PBF-EB) process was EIGA (Electrode Induction-melting Gas Atomization) atomized (Eckart TLS GmbH, Bitterfeld-Wolfen, Germany) using CMSX-4 master melt rods (Cannon Muskegon corporation, Muskegon, MI, USA). The powder fraction 45–105 µm was used for the experiments. The samples were fabricated on an Arcam A2 PBF-EB system (Arcam AB, Mölndal, Sweden) operating under Arcam EBM Control 5.2 Software at a build temperature of 1030 °C. 15 × 15 mm^2^ cuboids as well as 12 mm cylindrical samples were fabricated. A beam power of 360 W/300 W with a line offset of 150 µm/100 µm for block/cylinder geometry was used. The layer thickness was 50 µm. A standard Arcam cross snake hatching was applied and the scan pattern was rotated by 90° after each layer. For more information about the PBF-EB SX processing the reader is referred to the already published studies in this field^13,22^.

### Microstructural characterization

The samples were prepared for the microstructural investigations by means of a standard metallographic procedure consisting of cutting along the build direction (Brillant 220—ATM Qness GmbH, Mammelzen, Germany) and grinding with SiC paper down to 2500 as well as subsequent polishing (LaboPol-21—Struers GmbH, Willich, Germany) with 40 nm colloid SiO_2_ suspension. The texture of the samples was investigated by means of EBSD (Electron backscatter diffraction) technique using a NordlysNano detector (Oxford Instruments PLC, Abingdon, UK) operating in a Helios Nanolab DualBeam 600i FIB/REM (FEI Company, Hillsboro, OR, USA). An acceleration voltage of 25 kV as well as a beam current of 2.7 nA and a EBSD spot size of 5 µm under a specimen tilt angle of 70° was used. The IPF maps were generated using the software HKL Chanel 5. The open source Matlab toolbox MTEX 5.1.1 was used for the generation of the pole figures.

### Finite element simulation

A sequentially coupled thermo-mechanical finite element simulation was performed in Abaqus CAE 2018 (Dassault Systèmes Simulia Corp., Vélizy-Villacoublay, France) to investigate the emerging thermal stresses as a results of the induced temperature field. The beam path according to a standard snake like hatching for the calculation of the temperature field was implemented using a user subroutine of the Type DFLUX formulated in Fortran programming language. Here, the electron beam energy is incorporated as a standard Gaussian beam profile I(xyz,t) with a beam diameter σ of 400 µm as a function of time t and beam location x_B_, y_B_ and mesh size in Z-direction Z_Mesh_. The beam power P multiplied with the beam efficiency η is adapted according to the respective process parameters.

$${\mathrm{I}}\left({\mathrm{xyz}},{\mathrm{t}}\right)= \frac{{\mathrm{P}}\upeta }{2\pi {\upsigma }^{2}}{\mathrm{exp}}\left(-\frac{{({\mathrm{x}}-{x}_{B}({\mathrm{t}}))}^{2}+ {({\mathrm{y}}-{y}_{B}({\mathrm{t}}))}^{2}}{2{\upsigma }^{2}}\right)*\frac{1}{{Z}_{Mesh}}$$

A temperature independent specific heat capacity c_p_ of 705.1 J/gK as well as a latent heat ΔH_fus_ of 259.3 J/g was used^32^. The surrounding powder bed was implemented with a temperature independent density of 4900 kg/m^3^ according to the measurements of the powder tap density of 56% and the density of the bulk CMSX-4 Material of 8700 kg/m^3^. The build temperature was 1050 °C. The parameters for the subsequent calculation of the thermal stresses were chosen temperature dependent according to Table 1. The model assumes an isotropic, homogeneous material. The plastic deformation is calculated according to a yield criterion based on the von Mises stress.

**Table 1:** Temperature dependent properties used for the finite element calculation of the thermal stress field.

Temperature	Density	Young’s modulus	Poisson’s ratio	Thermal expansion	Yield stress e pl 0.2%	Yield stress e pl 1.5%	Thermal conductivity
Refs.	^32^	^33–35^	^35^	^36,39^	^17,37,38^	^17,37,38^	^32^
°C	kg/m^3^	GPa	–	K^−1^	MPa	MPa	W/mK
1000	8251	82.7	0.42	2.01E−05	448	550	22.3
1100	8206	75.1	0.43	2.10E−05	170	210	23.9
1200	8161	67.6	0.44	2.18E−05	44	44	25.8
1300	8116	60.1	0.45	2.27E−05	44	44	27.2
1320	8107	58.6	0.452	1.95E−05	44	44	27.2
1380	7754	54.0	0.458	1.86E−05	5	5	35
2000	7754	54.0	0.458	1.29E−05	5	5	35
